# Acceptability of the Dapivirine Vaginal Ring in Postmenopausal US Women

**DOI:** 10.1089/apc.2022.0002

**Published:** 2022-03-14

**Authors:** Mary Kate Shapley-Quinn, Nicole Laborde, Ellen Luecke, Craig Hoesley, Robert A. Salata, Sherri Johnson, Annalene Nel, Lydia Soto-Torres, Beatrice A. Chen, Ariane van der Straten

**Affiliations:** ^1^ Global Public Health Impact Center, RTI International, Berkeley, California, USA.; ^2^ Independent Consultant, San Rafael, California, USA.; ^3^ Department of Medicine, University of Alabama at Birmingham, Birmingham, Alabama, USA.; ^4^ University Hospitals Cleveland Medical Center, Case Western Reserve University, Cleveland, Ohio, USA.; ^5^ FHI 360, Durham, North Carolina, USA.; ^6^ International Partnership for Microbicides, Silver Spring, Maryland, USA.; ^7^ Division of AIDS, National Institute of Allergy and Infectious Diseases, Bethesda, Maryland, USA.; ^8^ University of Pittsburgh/Magee-Womens Research Institute, Pittsburgh, Pennsylvania, USA.; ^9^ Department of Medicine, Center for AIDS Prevention Studies, University of California, San Francisco, California, USA.; ^10^ ASTRA Consulting, Kensington, California, USA.

**Keywords:** pre-exposure prophylaxis, microbicides, dapivirine, intravaginal ring, acceptability and preference, postmenopausal

## Abstract

For women in the United States who remain sexually active beyond child-bearing years, susceptibility to HIV infection remains, yet condom use is low. We assessed acceptability of the dapivirine vaginal ring (ring) among 96 postmenopausal US women enrolled in a placebo-controlled multisite phase II trial of the ring, using questionnaires and in-depth interviews. Three quarters of women reported “perfect” adherence (ring never out) over the 3-month trial period. At study exit, the ring was found to be very easy to use by 72%, very comfortable to wear by 65%, and 4% reported it ever interfered with their daily activities. The most common worries among participants at preinitiation had decreased significantly at study exit (e.g., worries about inserting the ring declined from 46% to 6%, discomfort during daily activities from 53% to 3%, ring not staying in place from 48% to 14%, all *p* < 0.0001). Despite some couples feeling the ring during sex, the ring was perceived as more suitable than condoms for prevention because it was not burdensome to use, did not interfere with erection, and provided (for some) additional vaginal lubrication. The ring is a promising, highly acceptable HIV prevention method that is suitable to the lives of postmenopausal women and their male partners and can provide them with an additional prevention choice.

Clinical Trials Registration: NCT02010593.

## Introduction

Of the 1.5 million new HIV infections in 2020 globally, 50% were among women and girls.^1^ In the US, 17% of new infections in 2018 were among people older than age 50 years and of those, 27% of them were among women.^2^ The global population of people older than 50 years living with HIV is growing, including those infected before and after turning 50.^3^

Postmenopausal women are an important group for HIV prevention for several biological and behavioral reasons. Many studies have found that condom use declines with age.^4^ Notably, the 2008 National Survey of Sexual Health and Behavior found that condoms were not used “during most recent intercourse with 91.5% of casual partners, 76.0% of friends, 69.6% of new acquaintances, and 33.3% of transactional sexual partners” among people older than 50.^5^ Biological changes associated with menopause, like changes in the vaginal epithelium, also increase risk of HIV acquisition.^6–9^

In the US, daily oral pre-exposure prophylaxis (oral PrEP) is the only widely available product for HIV prevention apart from male and female condoms, although the recent US Food and Drug Administration (FDA) approval of Apretude (an injection to be administered every 2 months) will provide an additional option.^10^ Although studies show oral PrEP is >90% efficacious when used correctly,^11^ clinical trials and demonstration projects continue to show low uptake of oral PrEP in a variety of populations and settings.^12–14^ Among those who initiate oral PrEP, persistence beyond 6 months is low, and challenges with adherence result in lower than optimal effectiveness.^15–18^

The dapivirine vaginal ring (ring), developed by the International Partnership for Microbicides (IPM), resembles vaginal rings used for hormone delivery such as those used for management of menopausal symptoms (e.g., Estring^®^, Femring^®^). It slowly releases dapivirine (an antiretroviral drug) over a month-long period and is replaced monthly, thus minimizing user burden. Once inserted, the ring can be used discreetly and the month-long dosing period provides users with a long-acting method to reduce their HIV risk.^19^ In two phase III trials, the ring provided ∼30% reduction in HIV risk,^20,21^ two open-label extension trials estimated a protective level of 39%^22^ and 62%^23^ (respectively) for the ring, and modeling studies have suggested that the ring may be >50% effective in reducing HIV risk when used consistently.^24^

A global systematic review found vaginal rings to be well accepted for a range of indications.^25^ Recently, the World Health Organization (WHO) recommended use of the ring as an additional prevention choice for women at substantial risk for HIV infection. This is an important step toward offering worldwide more HIV prevention options that are controlled by women and provide longer periods of protection. The ring is going through the regulatory review process in several African countries.^26–28^

MTN-024/IPM 031 evaluated the safety and acceptability of the ring among postmenopausal women at three US sites.^29^ The primary goal of the trial was to collect safety data on the use of the ring among postmenopausal women. High overall acceptability and adherence to the ring (by self-report and corroborated by drug biomarker) and preference for the ring over male condoms were also reported.^29^ In this study, we sought to use quantitative and qualitative data to better understand postmenopausal women's attitudes and experience with ring use over time. The willingness and ability to use a ring among this age group is important, given the growing population of women older than 50 years who are sexually active, vulnerable to HIV, and with low condom use.

## Methods

### Study sample, design, and procedures

**FIG. 1.**
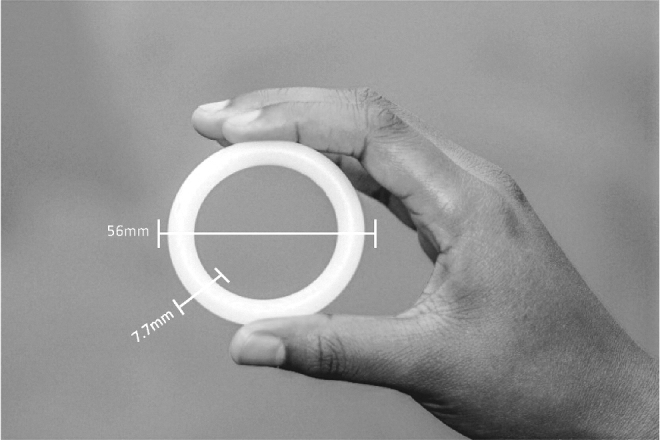
Dapivirine ring and dimensions.

MTN-024/IPM 031 was a Phase IIa multisite, randomized, double-blind, placebo-controlled clinical trial conducted at sites in Birmingham, Alabama; Pittsburgh, Pennsylvania; and Cleveland, Ohio. The study took place between December 2013 and April 2015. The active study product was a silicone elastomer vaginal ring containing 25 mg of dapivirine (Fig. 1). The placebo was visually identical to the active ring but did not contain dapivirine. Rings were inserted by the participant or a clinician at the study clinic, worn for a 4-week period, and replaced monthly for a total duration of 12 weeks. Women in the study were aged 45–65, healthy, postmenopausal (at minimum 12 months since last period or ≥6 months postbilateral oophorectomy), and HIV-uninfected.

Women were randomized in a 3:1 ratio to use the active or placebo ring, were instructed to use male condoms during any sexual intercourse for the duration of the study, and were given condoms and a vaginal lubricant. The use of other vaginal products was prohibited for the duration of the study. Further trial procedures have been previously reported.^29^ The study captured information regarding participants' motivation to join the trial, sexual behaviors and practices, partners, menopausal experiences, adherence to the ring, product preference and acceptability, and concerns about the ring. These data were collected at scheduled study visits, using computer-assisted self-interview (CASI) and case report forms for the full study sample, and using in-depth interviews (IDIs) at study exit in a subset of 24 randomly selected participants across all sites.

### Measures

CASI was administered to participants at their enrollment visit and at each follow-up visit (weeks 4, 8, and 12). The Menopause Rating Scale^30^ was CASI administered and assessed aging-related symptoms and their impact on women's quality of life. IDIs were conducted by a trained social scientist (N.L.) using WebEx video. The interviews were audio recorded and transcribed. Topics in the IDIs included experiences in the study, and with ring use, changes in sexual activity over the course of menopause, sexual experiences while using the ring and condoms, attitudes about the ring, perceptions of HIV risk, and future intentions to use the ring.

### Analysis

Descriptive quantitative data were summarized as mean/median (continuous variables) and tabulated (categorical variables) using Stata version 16.1. Change between baseline and exit visit in participants' reported worries related to ring use was assessed by the McNemar test.

The acceptability questionnaire and qualitative codebook were developed to align with the conceptual model described by Mensch et al.^31^ and adapted from a previous study of the dapivirine vaginal ring (MTN-020/ASPIRE). Qualitative interviews were coded thematically and analyzed using NVivo. Analysis of the qualitative data focused on attitudes and experience with the ring, including comments on insertion, removal, concerns, comfort and discomfort, and side effects. Further analyses were done to explicate findings that arose in the quantitative analysis, including differences in preferences between the ring and the condom.

### Ethics approval

The study protocol was reviewed and approved by the Institutional Review Board at each site, and the IRB at RTI International reviewed and approved the behavioral component. The study was overseen by the regulatory infrastructure of the Division of AIDS (DAIDS) and Microbicide Trials Network (MTN). All participants provided written informed consent. Those participants who completed the qualitative component confirmed their consent before beginning the IDI.

## Results

Ninety-six female participants were evenly recruited from the three study sites. The average age of study participants was 56.7 (range, 46–65). Participants identifying as White made up 66% of the sample, and 31% of participants identified as Black or African American. Further baseline characteristics of the study population and the qualitative subsample (*n* = 24) are described in Table 1.

**Table 1.** Baseline Demographic and Behavioral Characteristics of MTN-024 Study Participants

Baseline characteristics	Total* n* = 96	Birmingham* n* = 32	Pittsburgh* n* = 32	Cleveland* n* = 32	Qualitative* n* = 24
Mean age (range)^a^	56.7 (46–65)	56.5 (49–65)	57.7 (50–65)	55.9 (46–63)	56.2 (46–64)
More than a high school education	83%	75%	81%	94%	71%
Race^a^					
White	66%	50%	66%	81%	67%
African American/Black	31%	47%	31%	16%	33%
Biracial	3%	3%	3%	3%	0%
Ethnicity: Hispanic^a^	1%	0%	3%	0%	0%
Mean menopause symptom scale (range)	7.8 (0–22)	8.6 (1–22)	8.7 (0–22)	6.0 (0–16)	8.8 (1–22)
History of vaginal product use (female condom, vaginal ring, spermicidal sponge, cervical barrier, douche)	76%	81%	84%	62%	79%
History of tampon use	83%	81%	87%	81%	92%
Number of sex partners in the past 3 months					
0	38%	39%	42%	34%	42%
1	59%	58%	55%	63%	58%
2–3	3%	3%	3%	3%	0%
Sexual intercourse in past 30 days^a^	66%	75%	56%	66%	58%
Penile-vaginal sex	43%	38%	48%	44%	42%
Anal sex	2%	4%	0%	0%	0%
Receiving oral sex	30%	39%	29%	23%	41%
Giving oral sex	30%	33%	21%	33%	32%
Finger sex	23%	25%	19%	24%	10%
Nonpenetrative sex	42%	50%	24%	47%	29%
Male condom used during last sex act^b^	18%	16%	26%	13%	25%
Married or living with partner^a^	46%	38%	44%	56%	42%
Has a primary sex partner	62%	59%	61%	66%	63%
Length of relationship with primary sex partner					
Less than 1 year	11%	17%	6%	10%	7%
1–10 years	26%	44%	22%	14%	40%
11–20 years	63%	39%	72%	76%	53%
Primary sex partner is a man	97%	100%	100%	90%	100%
Primary sex partner experienced difficulty in sexual performance in past 3 months					
Never	47%	53%	42%	48%	27%
Ever	53%	47%	58%	52%	73%

^a^ Proportion of total sample previously reported in Chen et al.^29^ for select variables.

^b^ Among those who reported having had vaginal sex in their lifetime (*n* = 95).

### Sexual activity during menopause

Two-thirds of study participants had sexual intercourse in the 30 days before enrolling in the study; condom use during the last sex was low (18%). A variety of sexual relationships and engagement in different types of sex were reported (Table 1). Mean menopausal symptoms were mild and over half of those with a male partner reported that he had erectile dysfunction in the past 3 months. These findings, along with HIV risk perceptions reported qualitatively by participants, contributed to their assessment of rings and condoms for HIV/sexually transmitted infection protection at their stage in life.

### Ring use and experiences

Cumulative self-reported adherence to the ring was high, with 74% reporting that the ring was never out during the 3-month duration of the trial, and 91% reporting that it was never out for more than 12 h (Table 2). Six participants (7%) experienced the ring completely falling out on its own, primarily due to bowel movements or dislodging the ring when checking its position with her fingers. Fifteen participants (17%) removed the ring, mostly due to physical discomfort, the ring feeling out of place, worries about or not liking the ring, and wanting to clean it. Fifteen women (17%) experienced partial expulsions, in which the rings were pushed back into place without ever coming completely out. These events were caused by urinating, bowel movements, exercising, or checking the ring with the finger.

**Table 2.** Ring-Related Behaviors, Attitudes, and Perceptions-Overall and in the Qualitative Sample

	Total*n* = 94^a^	Qualitative*n* = 23^a^
Cumulative ring adherence (reported at weeks 4, 8, and 12)		
Ring was never out	74%	83%
Ring was never out for more than 12 h	91%	100%
Reasons for ring ever being out (asked by CASI)		
Full expulsion (ring came out on its own)	7%	0%
Ring removed	17%	14%
Partial expulsion (ring put back in place without removal)	17%	18%
Ring acceptability (reported by CASI at week 12)		
Ring never interfered with daily activities	96%	100%
Overall, how much do you like the ring?^b^	n* = 93*	n* = 23*
Like very much	37%	39%
Like	54%	52%
Dislike	10%	9%
Dislike very much	0%	0%
As a method to prevent HIV, which do you prefer to use?^b^	n* = 91*	n* = 20*
Ring	65%	65%
Condom	9%	13%
Both	24%	17%
Neither	2%	4%
Since you started the study, overall how easy or difficult was it to use the ring?^b^		
Very easy	76%	74%
Easy	23%	26%
Difficult	1%	0%
Very difficult	0%	0%
Since you started the study, overall, how did it feel to have the ring inside of you every day?^b^		
Very comfortable	65%	57%
Comfortable	32%	39%
Uncomfortable	2%	0%
Very uncomfortable	1%	4%
Vaginal ring is easy to insert^b^	85%	78%
Vaginal ring is easy to remove^b^	80%	78%
Perceived ring-associated changes in vagina	33%	43%
Vagina wetter	18%	30%
Vagina dryer	10%	4%
Problems experienced with ring		
Any physical discomfort	32%	22%
Any emotional discomfort	18%	13%
Any pain	13%	4%

^a^ Two of the 96 participants did not complete an exit CASI interview, one of whom was in the IDI subset.

^b^ Proportion of total sample previously reported in Chen et al.^29^ for select variables.

CASI, computer-assisted self-interview; IDI, in-depth interview.

### Acceptability

As shown in Table 2, and also previously reported,^29^ overall, the ring was highly acceptable with 91% reporting at study exit (3 months visit), they liked/very much liked the ring, 99% responding that the ring was “very easy/easy to use”, and another 97% responding that they were “very comfortable/comfortable with the ring inside every day.” Insertion and removal were also well tolerated, with 85% agreeing that the ring was easy to insert, and 80% agreeing that the ring was easy to remove.

Changes in the vagina when using the ring were reported by 33% of women. This included 18% who indicated that their vagina felt wetter (three-quarters were not at all bothered by it—data not shown) and 10% indicated that their vagina felt drier (one-third were not at all bothered by it; two-thirds were a little bothered—data not shown). Despite 32% reporting any physical discomfort and 18% reporting any emotional discomfort, only 4% said that the ring had interfered with their daily life.

**FIG. 2.**
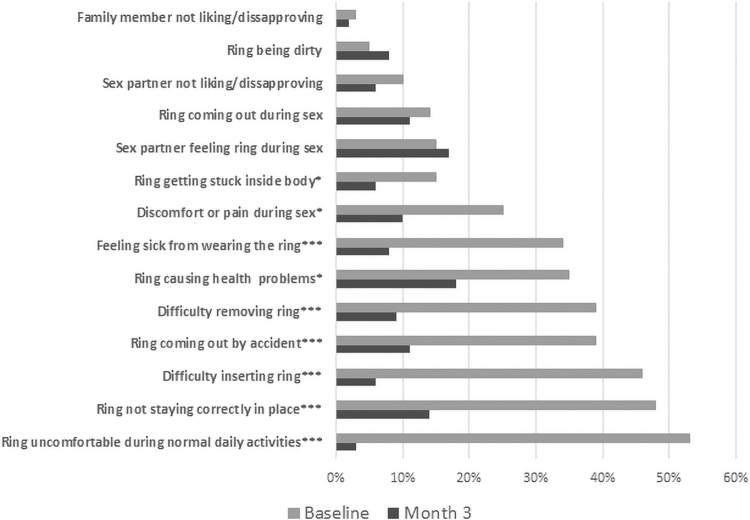
McNemar test: *p < 0.05, ***p < 0.0001. Changes in ring worries from baseline to month 3 (exit visit).

As shown in Fig. 2, at baseline (before using the ring the first time), a large proportion of participants had anticipatory worries about the ring, which decreased drastically between baseline and study exit (month 3). Of note, slightly more participants reported use-related concerns at month 3 than at baseline, including concerns about the ring being dirty and for “sex partner feeling the ring during sex” (Fig. 2). Different domains of acceptability are further explored below.

#### Sensation

Concerns about discomfort during normal daily activities decreased from 53% at baseline to just 3% at study exit. Consistent with these findings, most IDI participants expressed surprise that they could not feel the ring and were pleased that once it was inserted, “you can forget about it” (Cleveland, age 51). Another participant stated, “I was just, actually, blown away by the fact that I couldn't feel it at all…I thought it was going to feel like that but I didn't feel it at all” (Pittsburgh, age 53). Several participants reported that they could feel the ring shift during straining bowel movements but never experienced an expulsion. Two participants mentioned feeling the ring move and had concerns about the ring falling out.

#### Insertion and removal

Concerns about ring insertion (46%) and removals (39%) at baseline decreased to 6% and 9%, respectively, at study exit. Three participants reported actual inability to remove the ring. During IDIs, most participants did not describe issues with insertion or removal. Several participants were initially concerned about the size of the ring but found it easy to insert in practice. One participant commented that after the first insertion, she “became a champ at it” (Cleveland, age 58). There were a few exceptions: two women found ring insertion difficult due to their body size, and one found insertion difficult because of perceived swollen vaginal tissue after other study procedures were completed. A few participants spoke about their experiences with removal outside of the study clinic, but none of them reported challenges with removing the ring.

#### Side effects

As shown in Fig. 2, MTN-024 participants reported worries of the ring causing side effects at baseline, all of which were significantly lower at study exit. Worries that the ring would cause participants to feel sick were reported by 34% of participants at baseline and 8% at study exit, and worries that the ring would cause health problems were reported by 35% of participants at baseline and 18% at study exit. Qualitatively, most participants did not report side effects related to ring use during the study.

A few participants reported feeling fatigue, bloating, and/or nausea but were unsure if the symptoms were side effects of the ring or symptoms related to menopause. Other participants reported fewer hot flashes and less sweating related to menopause. Several participants reported increased vaginal lubrication, discharge, or “wetness.” This was not reported as problematic; in fact, one participant stated that increased lubrication made the vaginal examinations more comfortable, and another stated that the increased lubrication made sex more enjoyable for her.

### Prevention products and sex: relative preferences between condoms and ring

Many participants in the qualitative component assessed their own risk for HIV to be very low, in line with the recruitment criteria for this study. In keeping with this assessment, very few participants used condoms with their sexual partners before joining the study: only 18% of the total sample (and 25% of the qualitative subsample) used condoms during their last act of sex before starting the study.

The requirement of condom use in the context of the trial was challenging and unwelcome for most participants who had not used condoms recently, and some participants' male partners had difficulty maintaining an erection with the condoms. One participant said, “My husband is a little bit older than I am, and he had no interest whatsoever in wearing a rubber just because he's in his early ‘60s, and, you know, to get it up and to keep it up and, you know. So, basically for the three months [of the study duration] … We just totally, you know, worked around it” (Pittsburgh, age 54).

Participants shed light on the rare circumstances under which condoms were used outside the study context during the IDIs. A participant reported regularly using condoms for her protection because she had a partner who was living with HIV. Another participant practiced “safe sex” with her partner all the time and used condoms; they had been dating for less than a year. A third participant said she was “pretty forceful about using condoms after my divorce and I was sexually active.” (Pittsburgh, age 57).

**FIG. 3.**
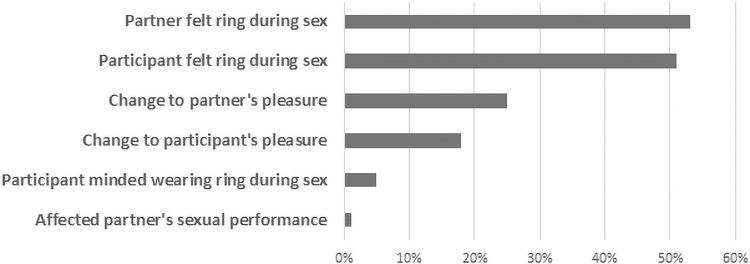
*n* = 47. Ring use experience during sex (month 3).

Most of the 47 sexually active participants did not mind wearing the ring during sex (95%), although 51% reported feeling it during sex and 53% reported that their sexual partner(s) felt the ring during sex (Fig. 3). As reported during the IDIs, feeling the ring was bothersome for some male partners, whereas others did not mind it. One participant whose partner found it bothersome explained, “No, he didn't like the idea of it [the ring] touching him. He could feel it, you know?” (Birmingham, age 55). This participant then used the ring as an excuse not to have sex with him for the remainder of the study, because she was not interested in being sexually active.

Another man felt the ring with his fingers during foreplay and asked his female partner to remove it. He had some sexual dysfunction, and they were rarely having intercourse. The one time they were going to engage in sex, he did not want the ring to be in place, so she removed it. On the contrary, one participant described her male partner feeling the ring when they had sex for the first time but not finding this problematic: “When he hit it—just for a second; that was it. It wasn't uncomfortable or anything” (Cleveland, age 51).

Several women interviewed indicated that the ring increased lubrication for them, but as noted earlier, this was a general sensation and was not in relationship to sexual experiences.

#### Ring versus condom preference

When asked about relative preference between condoms and vaginal rings, 65% of participants reported preferring the vaginal ring, whereas 24% liked both equally, 9% preferred condoms, and 2% preferred neither (Table 2). Among the participants who reported difficulties with or resistance to using condoms by their partner, many found the ring to be preferable as a prevention method. Participants who reported disliking condoms were more likely to select the ring as their preferred method (73%) compared to participants who reported liking condoms (54%, *p* = 0.01). Participants who liked condoms were also more likely to select “both” (41%).

In qualitative interviews, several women indicated that the ring provided greater control of protection during sex. Participants also expressed that the ring was more comfortable, easier to use, and allowed for more spontaneous sex. One participant articulated that “the ring would be tremendously better,” and “by far the better choice” compared with condoms (Pittsburgh, age 53).

Few participants who were not in a sexual relationship commented that they preferred the ring over condoms because it offered the opportunity to be in control of preventing HIV. Unlike condom use, ring use does not necessarily need to be discussed or negotiated ahead of time. One participant explained,

“I think for someone like me it would be great… what I like about it is it puts it on me to take of myself and I don't have to say, ‘Hey, put on a condom.’ You don't want to have that discussion. So, even though right now I'm not sexually active and maybe I never will be again but it's really good to know that I can control what happens to me which I think is a great thing” (Cleveland, age 64).

Multiple IDI participants described physiological barriers to maintaining sexual activity with their partners as they aged. These issues ranged from dementia and mental health challenges to postmenopausal symptoms and medications with side effects that affected sexual activity (erectile dysfunction for men, low libido). In addition to these issues, many participants cited condoms as an additional barrier to sex that was difficult to overcome. Providing a potential prevention method—like a vaginal ring—that is woman initiated and alleviates the need to be placed on the erect penis circumvented several challenges faced by those couples.

## Discussion

Participants enrolled in this study found the dapivirine vaginal ring to be highly acceptable and an appropriate option for their stage of life. Variations in the acceptability of the ring were linked to participants' worries about ring use, as well as their assessment of needing to use prevention products—like a vaginal ring and/or a condom—in their sexual relationships. Participant worries about the ring were frequent before ring initiation and most decreased significantly after 3 months of use, similar to findings among women of reproductive age in sub-Saharan Africa and the US.^32–34^ An analysis of adherence to the vaginal ring in the MTN-020/ASPIRE study showed that women who expressed worries about using the ring at baseline were less likely to use the ring consistently, indicating that addressing worries of potential ring users of all ages will be important for increasing adherence.^35^

The reduction of worries over time and with experience using the vaginal ring supports findings from studies with reproductive age women showing that familiarity (or lack thereof) with the ring has important implications for user uptake and acceptability.^25,36^ For PrEP-naive women of reproductive age, a US study showed that even experience with an analogous contraceptive product may help overcome familiarity barriers: preferences among 10 potential PrEP delivery forms were driven by a desire for comfort and ease of use and linked to experience with a similar delivery form for contraception.^37^ Among these participants, those who had previously and were currently using a vaginal ring for contraception were more than five times as likely to choose a vaginal ring for PrEP compared to those who had never used a vaginal ring.

The responsibility of assessing potential ring users' (of any age) familiarity with the product and worries before initiation—and providing appropriate counseling in response—will fall to health care providers and allied health professionals, indicating a need for education, training, and support for these providers. On the user side, it will be critical to normalize this “novel” vaginal delivery form, build trust, and mitigate the spread of misinformation to avoid negative effects on ring awareness and uptake, as seen previously for insertable contraceptive methods and tampons.^38,39^

While a growing body of literature describes how male partner relationships, power dynamics, and sex practices may impact ring use for adolescent girls and young women,^40–42^ our findings provide important considerations for postmenopausal women. A majority of women in this study preferred vaginal rings over condoms for HIV prevention, as was true for the reproductive-aged participants in a large Phase III trial of the same vaginal ring who compared the study product to eight other possible HIV prevention products.^19^ However, the age-related barriers to maintaining an active sex life raised by these participants (postmenopausal symptoms, male partner erectile dysfunction, low libido) often aligned with an aversion to condom use and highlighted an important additional layer of considerations unique to older women.^36^

Like their younger counterparts, MTN-024 study participants also described an appreciation for a user-controlled HIV prevention method that women could initiate, avoiding the need for negotiation or discussion with sexual partners, and experience with the ring during the 3-month trial provided plenty of time to become comfortable with the delivery form and overcome initial worries about use.

Important limitations of this study include the relatively low risk profile of recruited participants (as determined by the recruitment criteria for participants), small sample size, and the potential for social desirability in participant responses. Only 66% of participants were sexually active in the month before joining the study, and only 45% had engaged in vaginal or anal sex in that time. While concerns have been noted regarding the limited ability of vaginal microbicides (including the ring) to provide protection from HIV exposure during receptive anal intercourse,^43,44^ behavioral data collected in MTN-024 was not positioned to understand the context of HIV prevention among postmenopausal women who may engage in anal sex, as only one participant reported recently engaging in anal sex at baseline, and this participant was not selected for the qualitative component.

Qualitatively, very few participants described situations where they felt at risk of HIV in their sexual encounters (i.e., dating after a divorce or in a relationship with someone living with HIV). For the majority who perceived themselves to be at low risk of HIV, assessment of acceptability may differ from postmenopausal women who see themselves as at higher risk of contracting HIV. The qualitative data presented here are drawn from a random subset of women in the larger clinical trial. Despite the small sample size, interviews reached saturation on key themes. Finally, being prospectively followed in a 3-month clinical trial of the ring, participants may have exhibited some social desirability when responding to questions about product worries at the end of the study, and in discussing their views of the ring during interviews.

In conclusion, for women who remain sexually active beyond child-bearing years, susceptibility to HIV infection remains, yet, condom use is low. The dapivirine vaginal ring is a promising, highly acceptable HIV prevention method that can provide an additional choice for postmenopausal women.
